# Physical activity associates with subarachnoid hemorrhage risk– a population-based long-term cohort study

**DOI:** 10.1038/s41598-019-45614-0

**Published:** 2019-06-25

**Authors:** Joni V. Lindbohm, Ilari Rautalin, Pekka Jousilahti, Veikko Salomaa, Jaakko Kaprio, Miikka Korja

**Affiliations:** 1 Clinicum, Department of Public Health, University of Helsinki, P.O. Box 41, FI-00014 Helsinki, Finland; 2 Department of Neurosurgery, University of Helsinki and Helsinki University Hospital, P.O. Box 266, FI-00029 Helsinki, Finland; 3 National Institute for Health and Welfare, P.O. Box 30, FI-00271 Helsinki, Finland; 4 Institute for Molecular Medicine FIMM, P.O. Box 20, FI-00014 Helsinki, Finland

**Keywords:** Risk factors, Neurology

## Abstract

Benefit of physical activity in prevention of aneurysmal subarachnoid hemorrhage (SAH) is unclear. We aimed to clarify this by studying how different types of physical activity associate with SAH risk. By following 65 521 population-based FINRISK participants prospectively from medical and autopsy registries since 1972 until 2014, we detected 543 incident SAHs. At baseline, we measured leisure-time physical activity (LTPA), occupational physical activity (OPA), and commuting physical activity (CPA) levels. The Cox model adjusted for all well-known SAH risk factors and for socioeconomic status, provided hazard ratios (HRs) for physical activity variables. Every 30-minute increase in weekly LTPA decreased SAH risk linearly in men and women HR = 0.95 (95% CI = 0.90–1.00). CPA reduced SAH risk as well, but the association diminished as participants retired. In contrast, individuals with moderate (1.41, 1.04–1.92) and high OPA (1.34, 0.99–1.81) had elevated SAH risk. Protective association of LTPA persisted in all age and hypertension groups, and was even greater in current smokers 0.88 (0.81–0.96) than non-smokers (p = 0.04 for difference). Commuting and leisure time physical activity seem to reduce SAH risk in men and women and is most beneficial for smokers. Future intervention studies should investigate whether physical activity can reduce the rupture risk of intracranial aneurysms.

## Introduction

Aneurysmal subarachnoid hemorrhage (SAH) has remained a serious disease with a case fatality of approximately 40%^1,2^. In development of SAH, lifestyle risk factors play the main role^3^. The risk factors include smoking, hypertension, increasing age, and possibly adverse lipid profile and female sex^4–9^.

Leisure-time physical activity (LTPA) protects against ischemic stroke^10,11^ but only few studies exist on SAH and physical activity^12–19^ with inconsistent results. Recently, a study^19^ combining all physical activity types suggested that high overall physical activity elevates SAH risk. However, the study did not differentiate associations by physical activity type, even though these types may associate differently with cardiovascular diseases^10^. Our aim was to study whether LTPA, commuting physical activity (CPA), and occupational physical activity (OPA) decrease SAH risk and whether the effects distinct by different types of physical activities.

## Results

Between 1972 and 2014 the follow-up included 65 521 participants and provided 1.52 million person-years. There were 543 incident SAHs (215 men), of which 98 were fatal outside the hospital or in emergency rooms. Of the whole cohort, 24.3% used motorized transport for commuting and 18.5% had low occupational activity (Table 1). These proportions did not differ significantly by sex.

**Table 1:** Baseline characteristics of cohort by SAH status.

Variable	No. SAH	SAH
Participants, n	64 835	543
Age at baseline, years, mean (SD)	45.3 (12.1)	46.0 (11.5)
Sex, men (%)	48.40%	46.20%
BMI (kg/m^2^)	26.3 (4.4)	26.1 (4.2)
Blood pressure, mean (SD)		
• Systolic (mmHg)	140.9 (21.4)	147.1 (23.7)
• Diastolic (mmHg)	84.6 (12.8)	89.3 (12.7)
Smoking, n (%)		
• Never	34 670 (53.5)	235 (43.3)
• Former	11 736 (18.1)	80 (14.7)
• Current	17 337 (26.7)	215 (39.6)
• Missing data	1 092 (1.7)	13 (2.4)
Alcohol, g/week, mean (SD)	53.6 (96.6)	68.1 (115.3)
Total cholesterol, mmol/l, mean (SD)	6.0 (1.3)	6.3 (1.4)
Education, n (%)		
• Low	18 089 (27.9)	162 (29.8)
• Moderate	21 858 (33.7)	186 (34.3)
• High	23 464 (36.2)	180 (33.1)
• Missing data	1 424 (2.2)	15 (2.8)
Leisure-time physical activity, n (%)		
• Low	19 822 (30.6)	184 (33.9)
• Moderate	32 365 (50.0)	281 (51.7)
• High	11 391 (17.6)	62 (11.4)
• Missing data	1 257 (1.9)	16 (2.9)
Commuting physical activity, minutes, n (%)		
• No work	17 453 (26.9)	156 (28.7)
• Motor transport	15 789 (24.4)	116 (21.4)
• <30	17 208 (26.5)	147 (27.1)
• ≥30	9 618 (14.8)	88 (16.2)
• Missing data	4 767 (7.4)	36 (6.6)
Occupational physical activity, n (%)		
• No work	18 100 (27.9)	160 (29.5)
• Low	12 038 (18.6)	67 (12.3)
• Moderate	13 744 (21.2)	122 (22.5)
• High	18 045 (27.8)	168 (30.9)
• Missing data	2 908 (4.5)	26 (4.8)

### LTPA

**Figure 1:**
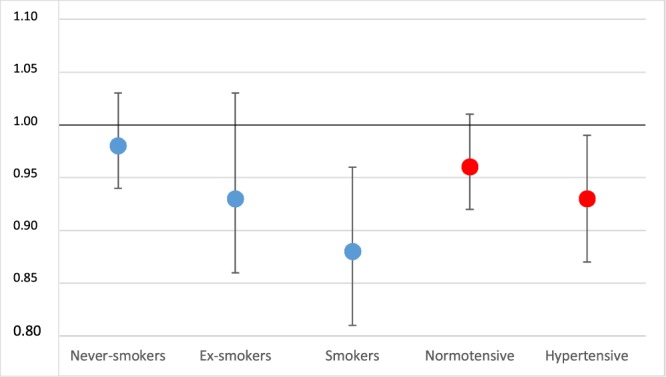
Every 30-minute increase in weekly leisure time physical activity reducing subarachnoid hemorrhage risk more in smokers and ex-smokers than in never-smokers (p = 0.04), with no difference between hypertensives and normotensives (p = 0.29). Y-axis describes HR and the points describe point estimates, the whiskers describe 95% CIs.

Every 30-minute increase in leisure-time exercise (leading to mild sweating and mild breathlessness) per week associated linearly with a decreased SAH risk of 5% in both men and women (Table 2). This association remained the same even 40 years after answering the questionnaire. This association also persisted in all SBP and age groups, and remained the same after OPA and CPA were included in the model. The protective association of every 30-minute increase in weekly exercise was stronger among smokers (0.88, 0.81–0.96) and ex-smokers (0.93, 0.86–1.03) in comparison with never-smokers (p = 0.04) (Fig. 1). In line with this, smokers with less than 90 minutes of LTPA per week had a relative excess risk (RERI) of 2.03 (0.10–3.96).

**Table 2:** Hazard ratios for subarachnoid hemorrhage by leisure-time- and occupational physical activity for all subarachnoid hemorrhage cases and separately by sex.

	No. of SAHs (Overall)	HR (95% CI)	No. of SAHs (Men)	HR (95% CI)	No. of SAHs (Women)	HR (95% CI)
Occupational physical activity						
Low	67	1	28	1	39	1
Moderate	122	1.41 (1.04–1.92)	53	1.66 (1.04–2.64)	69	1.23 (0.81–1.85)
High	168	1.34 (0.99–1.81)	102	1.40 (0.90–2.16)	66	1.26 (0.82–1.94)
p-for linearity departure		0.10		0.06		0.60
Weekly LTPA time (per each 30-minute increase)	203	0.95 (0.91–0.98)	100	0.95 (0.90–1.00)	103	0.95 (0.90–1.00)

Model for OPA include only employed participants. All models adjusted for age, sex, study year, study area, BMI, systolic blood pressure, cholesterol, and smoking.

According to the PAF analysis, a weekly rise in LTPA from 0 to >90 minutes among inactive smokers associated with, 11% decrease in smokers’ SAH events. In comparison, the same analysis showed that diminishing hypertension in smokers associated with similar magnitude of 9% decrease in SAHs.

### CPA

**Figure 2:**
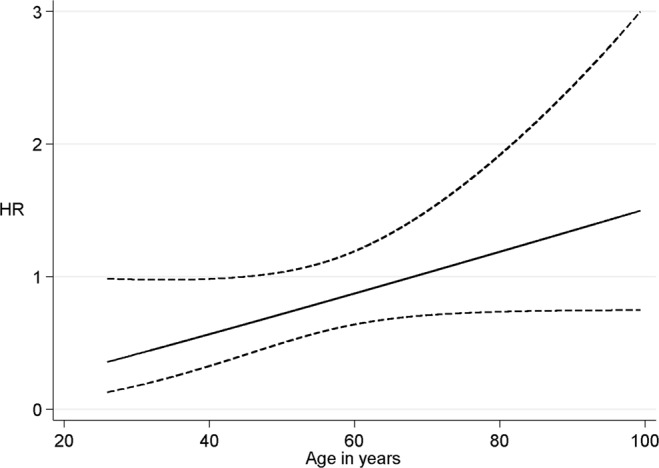
Change in HR and 95% CI for high commuting physical activity by age in years. Protective association of HR for high commuting physical activity diminishes as participants approach retirement age.

**Figure 3:**
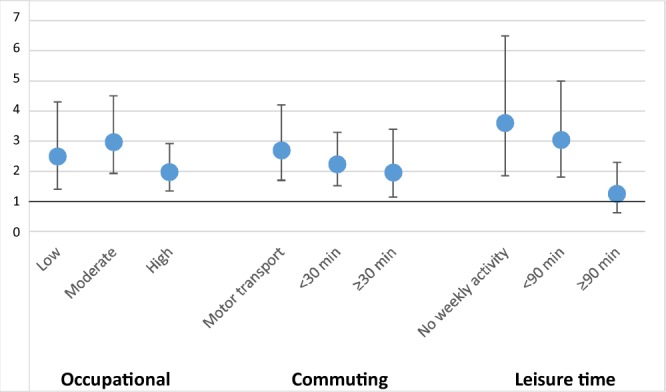
Associations between smoking and SAH in all three physical-activity groups. High commuting- and leisure-time physical activities seem to counteract risk increasing association of smoking. Y-axis describes HR and the points describe point estimates, the whiskers describe 95% CIs.

**Figure 4:**
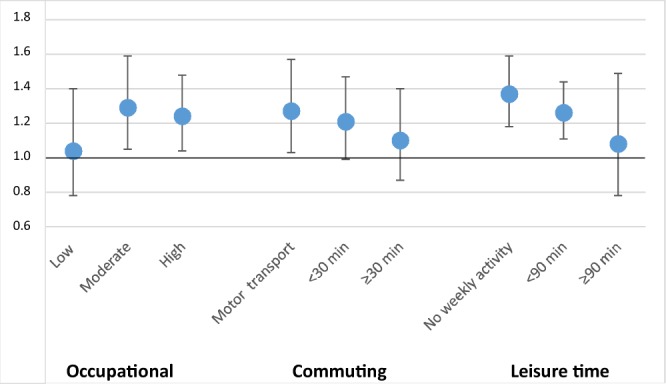
Associations between 1 SD increase (21.4 mmHg) in SBP and SAH in all three physical- activity groups. Y-axis describes HR and the points describe point estimates, the whiskers describe 95% CIs.

Active commuter (≥30 minutes per day) had reduced SAH risk when compared to those who used motorized transport. This reduced risk persisted until participants reached retirement age (Fig. 2). The SAH risk associated with smoking decreased in active commuters when compared to the low-CPA group (Fig. 3). Similarly, an increase in SBP (standard deviation increase of 21.4 mmHg) did not associate with SAH risk in active commuters (Fig. 4).

### OPA

In contrast to LTPA and CPA, an adjusted model including both sexes showed that moderate OPA elevated SAH risk and high OPA may elevate SAH risk (Table 2). In subgroup analysis, moderate OPA was associated with elevated SAH risk only among female smokers. In this group of women, an additive interaction emerged between female heavy smokers (over 10 cigarettes per day) and moderate OPA with RERI of 2.92 (0.30–5.53). Moreover, every SD increase (21.4 mmHg) in SBP was associated with elevated SAH risk in individuals doing physical work (high- and moderate-OPA groups) but not in the low-OPA group.

### Competing risks analysis

When weekly LTPA in minutes was divided into quartiles, SAH risk decreased with increasing physical activity also in presence of competing risk of death (Supplementary Fig. 1). No differences in HRs emerged between sudden-death SAH and hospitalized SAH patients in any physical activity category.

## Discussion

Every 30-minute increase in weekly LTPA, such as brisk walking, swimming, cycling, or jogging, associated with 5% reduced SAH risk in both men and women. In theory, by increasing LTPA by up to 5 hours a week, one could reduce the risk of SAH by 40%. The protective association of LTPA remained the same not only in all age groups but also in all SBP groups, suggesting that presence of SAH risk factors does not cancel out the protective association of LTPA. Interestingly, the protective association of LTPA was even stronger among ex-smokers and smokers. In fact, smokers with less than 90 minutes of LTPA per week had a relative excess SAH risk of 203%. Thus, motivating smokers to be physically more active, not only quitting smoking, may be both a new and a promising approach in preventing SAH.

Similar to LTPA, high commuting activity, often by foot or by bicycle, associated with reduced SAH risk in men and women and this association persisted until retirement age. Interestingly, smoking and hypertension, the most important SAH risk factors^5,6^, increased the risk of SAH less in individuals with moderate or high CPA than in individuals with low CPA. In other words, similar to LTPA, CPA may counteract the hazardous effects of smoking and hypertension. The reasons behind the potential protective effect of LTPA and CPA remain speculative but aerobic physical activity reduces systemic inflammation^20^ and can also reduce inflammation in intracranial vessel walls^21^. Our finding on LTPA and CPA are further supported by studies on myocardial infarction and physical activity that have shown a decreased risk in high LTPA^10,11^ and CPA^11^ groups.

Moderate OPA (work including much walking but not lifting heavy objects) and high OPA (work that includes much walking and frequent lifting of heavy objects or climbing stairs) associated with elevated SAH risk when compared to office work (low OPA). These findings are also consistent with studies on myocardial infarct and stroke^10,22,23^. The reasons behind elevated SAH risk in moderate and high OPA groups may relate to different health behavior regarding hypertension and smoking in different OPA groups as our results to some extent suggest. Other explanations include residual confounding from inaccurate SES-variable, or an unknown work-related external risk factor. In terms of the high-OPA, the elevated risk may also stem in part from differences in health effects between high OPA and high LTPA^23^. One possible explanation for harmful effects of OPA in SAH risk may be the different nature of physical activities. LTPA and CPA are relatively short term aerobic cardiovascular exercises, whereas OPA may be more long term and consists more often of lifting of weights, thus resembling anaerobic weight training. The prolonged physical stress in high OPA group may thus become harmful if the workload surpasses the limits of individual’s cardiorespiratory system and may even act as trigger of cardiovascular diseases^22,24^. Therefore, also cardiovascular effects between LTPA, CPA and OPA may differ. However, in our observational study we can only speculate the possible causal mechanisms between OPA and SAH and further intervention studies on this topic is needed.

A recent study^19^ suggested that when OPA, CPA, and LTPA were combined into a continuous variable of MET hours per day, high physical activity (>33 MET hours) elevated SAH risk. The 33 MET hours correspond to ~4.5 hours of jogging (LTPA) per day, whereas people in moderate and high OPA groups reach 33 MET hours during a normal working day. Therefore, the elevated SAH risk observed in the Japanese study^19^ may reflect elevated SAH risk in the moderate and high OPA groups – not harmful effects of physical activity per se.

To our knowledge, only two studies on physical activity and SAH exist that include sudden deaths from SAH and control properly for confounding factors^16,17^. The current study is an update of a study by Hu *et al*.^17^ published in 2005 and describes similar results. Our updated data set includes a longer follow-up, more participants, more physical activity variables, and twice as many first-ever SAH cases, thus providing more detailed, reliable, and novel results. A Norwegian study^16^ found no association between physical activity and SAH in an analysis including 108 SAHs, although their HR trends for physical activity were similar to ours.

Because association of physical activity with SAH seems less strong when compared to classical SAH risk factors, namely smoking and hypertension, the number of SAHs needs to be relatively high when studying physical activity.

The study may have a few strengths. We analyzed physical activity in three different types of activity categories, enabling us to perform the most detailed analysis of physical activity and SAH to date. The follow-up of up to 40 years is one of the longest among cardiovascular risk factor studies^25^. The relatively large number (543) of first-ever SAH cases enabled reliable subgroup analyses. Our prospective set-up reduced the risk of informational bias and reverse causality. Our analysis included SES, the known risk factors of SAH, and outside-hospital deaths from SAH with high diagnostic accuracy^26^. The majority of physical activity studies^12–15,18^ have not included the substantial fraction of SAH patients dying suddenly outside hospitals. Though sudden-death SAH individuals have worse risk-factor profiles than do hospitalized SAH patients^27^, we found no differential effect of any physical activity categories on the two types of outcome. As the Finnish incidence of SAH does not differ from the incidence found in other countries reporting reliable (autopsy reports included) incidence rates^28^, our results may be generalizable at least to other Caucasian populations.

The study has also limitations. Even though our positive predictive value for aneurysmal SAHs was high, our dataset is likely to include small proportion of non-aneurysmal SAHs (perimesencephalic bleed, arteriovenous malformation, fistula, tumor, or trauma related SAHs). However, given the high positive predictive value of aneurysmal SAH diagnoses, and lack of robust association between physical activity and these non-aneurysmal SAHs, they are unlikely to have major contribution to our results. Our study is observational and strong inference about causality cannot be made. Participants’ risk factor profiles were collected at enrolment, and therefore changes in risk factors during the follow-up have evidently taken place. However, we were able to take into account the changing effects of CPA during follow-up; other variables did not show strong evidence of changes. We measured physical activity by questionnaire, and responses are prone to recall and reporting bias. However, our questionnaire has been validated against accelerometer^29^ and mortality and morbidity data^11^. Our questionnaire-measured physical activity correlates well with mortality and morbidity whereas the correlation with accelerometer-measured physical activity revealed a moderate correlation. In accelerometer study, physical activity was consistently over-reported in the questionnaires^29^, suggesting that our results likely underestimate the potential benefits of LTPA and CPA.

In conclusion, increases in leisure-time and commuting physical activity are likely to reduce SAH incidence in all age- and SBP groups and especially among smokers. Increasing commuting and leisure time physical activity could be a novel non-invasive preventive method for reducing the rupture risk of intracranial aneurysms. However, intervention studies are needed to confirm this. The reasons and mechanisms for elevated SAH risk in those doing strenuous physical work remain to be studied.

## Methods

Previous studies describe the research protocol in detail^1,5,25^. The National FINRISK Surveys, conducted every five years since 1972, collected data on independent, population-based, random samples of adults from various geographical areas of Finland. The participation rate in FINRISK surveys in the 70’s and 80’s was >90% and 80% and has remained over 60% in all surveys^25^. The questionnaire measured physical activity in three main domains of daily activity: CPA, OPA, and LTPA. The analysis of CPA and OPA included only those who were employed. Supplemental Methods describes the data collection, physical activity, and other variables in detail. The local ethics committees at the University of Helsinki and Helsinki University Hospital gave approval for each FINRISK survey in accordance with legislation pertinent to the time of the survey. The World Medical Association’s Declaration of Helsinki on ethical principles for medical research also guided the studies. Each participant provided oral informed consent between 1972 and 1997, whereas from 2002 onwards, participants also gave a written informed consent^25^.

### SAH identification and definition

Follow-up started at enrolment and ended at first-ever SAH, death, or on December 31, 2014, whichever came first. The follow-up was complete for deaths and hospitalization when the subject was resident in Finland; those moving abroad were censored at the time of emigration^1^. The nationwide Hospital Discharge Register and Causes of Death Register identified nonfatal and fatal SAHs with high accuracy^30^. Sudden deaths from SAH occurred away from hospitals, in an ambulance, or emergency room and were confirmed in forensic autopsy, medical autopsy, or with clinical or radiological examinations (spinal tap and/or computer tomography), or both. Externally validated positive predictive value of overall aneurysmal SAH diagnoses was 87%, whereas it was 97% for sudden-death aneurysmal SAHs^30^. The strengthening the Reporting of Observational studies in Epidemiology (STROBE) statement^31^ guided the reporting.

### Statistical analyses

Scatter plots and Spearman’s correlation coefficients studied correlations between physical activity variables measured by the questionnaire. The Cox proportional hazard model calculated hazard ratios (HRs) between physical activity and SAH while Schoenfeld residuals and log-log plots examined proportionality assumptions. Due to long follow-up, we also calculated a competing risks model^32^. Based on the SAH literature^4–7^, the final adjusted model included all known SAH risk factors, (age, sex, SBP, smoking) and one to three physical activity variables. To control the cohort effect and potential confounding, we also included BMI, cholesterol, study year, and study area into the final model. Preliminary models also included SES and alcohol consumption. The likelihood-ratio test examined multiplicative interactions, and cubic splines examined linearity. Additive interactions^33^ were modelled as relative excess risk (RERI). To avoid over-estimation of population-attributable fraction (PAF), these were calculated by the average attributable fraction method, which restricts overall PAFs to 100%^34^. Analysis plan in the Supplementary File describes the predefined analysis protocol. All statistical analyses used Stata Corp version 14.2 (Stata Corp, College Station, TX).

## Supplementary information

Supplementary file
