# Supplementary material for: Physical activity associates with subarachnoid hemorrhage risk– a population-based long-term cohort study
Source: Sci Rep. 2019 Jun 25;9:9219. doi: 10.1038/s41598-019-45614-0 (PMC6592878; doi:10.1038/s41598-019-45614-0)
Supplement: Supplementary file 1 — Supplementary file [file 41598_2019_45614_MOESM1_ESM.pdf]

Physical activity associates with subarachnoid haemorrhage risk– a population-based long-term cohort study

*Joni V. Lindbohm, MD, PhD;[1,2] Ilari Rautalin, BM;[1,2] Pekka Jousilahti, MD, PhD;[3] Veikko Salomaa, MD, PhD;[3] Jaakko Kaprio, MD, PhD;[1,4] Miikka Korja, MD, PhD;[2]

[1]Clinicum, Department of Public Health, University of Helsinki, P.O. Box 41, FI-00014 Helsinki, Finland

[2]Department of Neurosurgery, University of Helsinki and Helsinki University Hospital, P.O. Box 266, FI-00029 Helsinki, Finland

[3]National Institute for Health and Welfare, P.O. Box 30, FI-00271 Helsinki, Finland

[4]Institute for Molecular Medicine FIMM, P.O. Box 20, FI-00014 Helsinki, Finland

Correspondence to Joni Lindbohm, Department of Public Health, University of Helsinki, P.O. Box 41, FI-00014 Helsinki, Finland; E-mail address: joni.lindbohm@helsinki.fi; Telephone: +358 9 1911 Fax: +358 9 191 27 600

Supplementary file

Supplementary Figure 1. Competing risks regression for weekly leisure-time physical activity levels in quartiles. Other causes of death are competing risks and y-axis describes cumulative (increasing) incidence.

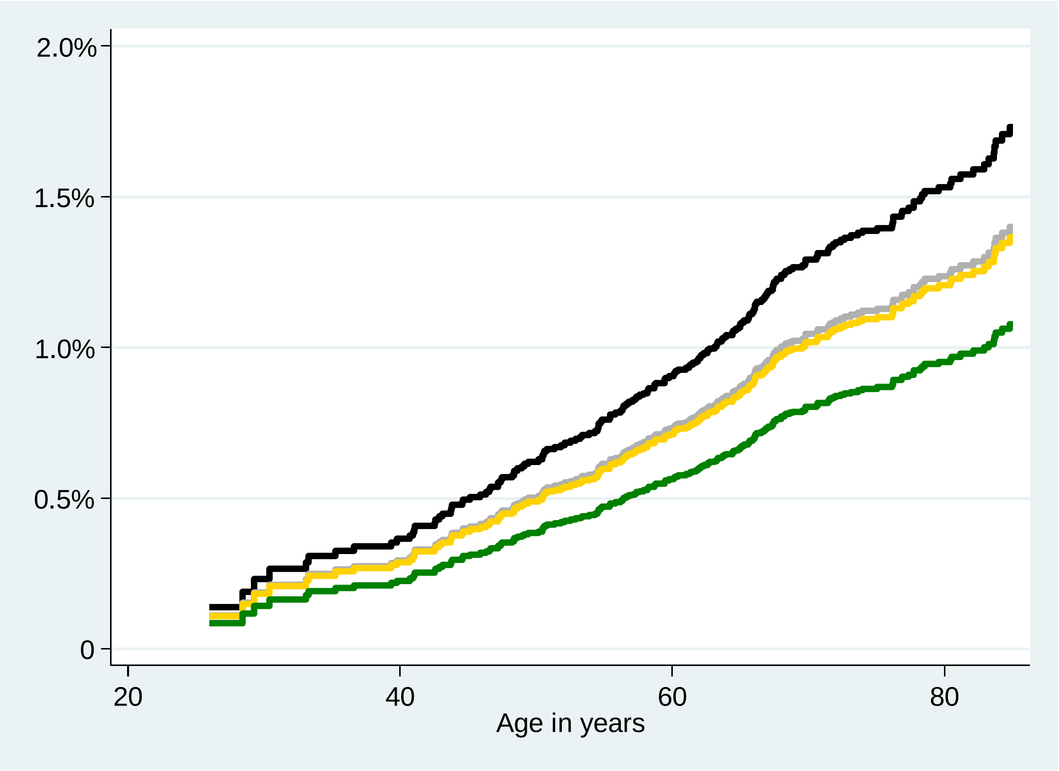

Black: <22 min of weekly activity, gray: 22-89; yellow: 89-178; green: >178 min.

Supplementary methods

Physical activity variables

At enrollment, physical activity was measured by questionnaire in three main domains of daily activity: commuting physical activity (CPA), occupational physical activity (OPA), and leisure-time physical activity (LTPA). Questions on CPA, OPA, and LTPA have served to measure physical activity in each FINRISK survey since the beginning of the follow-up (1972).

LTPA was measured with three questions at enrollment. The first question concerned the quality of leisure-time physical activity with four response options: (1) low (mostly inactive, e.g. reading and watching television), (2) moderate (at least 4 hours of exercise weekly, e.g. walking, fishing, hunting, or light gardening), (3) high (e.g. running, skiing, swimming, or more vigorous sports more than 3 hours per week), (4) very high (competitive exercising almost daily). Based on the literature and number of SAHs in these categories, we reduced these to three categories by combining options 3) and 4).

In the FINRISK Surveys, CPA was divided into six categories: (1) not working or using motor transport to commute, and active daily (2) less than 15 minutes (min), (3) 15-29 min, (4) 30-44 min, (5) 45-59 min, or (6) over 60 min commuting. For analyses of CPA and OPA, we excluded those who did not work. Furthermore, based on number of SAHs, we used the following classification: 1) motor transport only, 2) <30 min, 3) ≥30 min per day.

For OPA, FINRISK participants chose the best description of his/her job at enrollment from four options: (1) mainly sedentary work, (2) work including much walking but not lifting heavy objects, (3) work that includes besides walking, much / frequent lifting heavy objects and climbing stairs for example, (4) work which is mainly manual labor. Based on the literature and number of SAHs, we used three categories for OPA: 1) low, 2) moderate, and 3) high/heavy.

The other two FINRISK questions about characteristics of LTPA since 1982 were: 1) How many times do you usually exercise (at least mildly sweating) per week, and 2) How long does your leisure time physical activity last each time. Question 1 was continuous, but question 2 was categorical with five different answer options: (1) 0 min, (2) <15 min, (3) 15-29 min, (4) 30-59 min and (5) ≥60 min. Based on these two questions, we created variables describing weekly LTPA activity in minutes by multiplying the average of each category in question 2 by that in question 1. For the over-60-minute LTPA-active group in question 2, we used 90 min of LTPA activity because the average was not calculable. The resulting continuous variable was then scaled to provide a unit increase in weekly LTPA activity per 30 min

To describe participants' total physical activity, we created an overall physical activity (PA) index by combining our three main physical activity variables (CPA, OPA, and LTPA). Based on the literature, the PA index was categorized in four different categories: (1) inactivity, when all three response options of the questions were 1 (inactive/least active) (in the questionnaire), (2) low activity when response options were 1-3 in CPA; 1-2 in OPA, and 1 in LTPA, but were not included in the inactive category, (3) moderate activity when response options were 4 in CPA, 3 in OPA, and 2 in LTPA, and (4) high activity when response options were 5-6 in CPA, 4 in OPA, and 3-4 in LTPA. Based on number of SAHs, we combined groups 1 and 2.

Other variables

The population register of Finland provided information about participants' age and sex. At enrollment, experienced and specialized nurses did clinical measurements including systolic blood pressure (SBP), height, and weight, and acquired semi-fasting blood samples for cholesterol measurement after at least 4-hour fasting. A standardized self-administered questionnaire gathered data on health and lifestyle, including alcohol consumption, history of hypertension, physical activity, smoking status, and socio-economic status (SES).

SBP, cholesterol, and body mass index (BMI) served as continuous variables in our analyses. BMI was calculated as weight in kilograms divided by squared height in meters. Participants' smoking status was divided into three categories: 1) non-smokers, 2) former smokers (quit >6 months ago), and 3) current smokers. Individuals with a history of hypertension or medication for hypertension or BP >140/90 mmHg received the classification hypertensive.

Analysis plan in brief

Power calculations

Power calculations for subgroup analyses based on incidence estimate of 20/100 000, p=0.05, power=0.80, and estimated clinically meaningful effect of HR 1.10 per standard deviation increase in continuous variables and HR 1.20 in categorical variables.

Included confounders

Confounder included based on literature[1-4]: age, sex, SBP, smoking and one to three physical activity variables.
To control the cohort effect and potential confounding: BMI, total cholesterol, socioeconomic status, alcohol consumption, study year, and study area were included in analyses.
Multi collinearity examined with Scatterplots and Spearman´s correlation coefficient.

Analysis model performed separately for each physical activity type and physical activity index

Main analysis: Cox proportional hazards model. Proportionality assumption examined with Schoenfeld residuals and log-log plots. Informative censoring and competing risks examined with Fine and Gray competing risks model.[5] Non-linear associations examined with cubic splines and multiplicative interactions examined with likelihood ratio test. Additive interactions examined as relative excess risk (RERI)[6] and population attributable fraction estimated with average attributable fractions method to avoid overestimation.[7]

Analyses done that were not included in the original analysis plan

Sex stratified multiplicative interaction and additive interaction test in the occupational physical activity group. The original analysis plan aimed to study these interactions only in the group including men and women because of expected limited power in sex specific analyses. However, because of somewhat surprising finding on occupational physical activity we studied the interactions also by sex.

References:

1 Lindbohm, J. V., Kaprio, J., Jousilahti, P., Salomaa, V. & Korja, M. Sex, Smoking, and Risk for Subarachnoid Hemorrhage. Stroke 47, 1975-1981, doi:10.1161/STROKEAHA.116.012957 (2016).
2 Korja, M. et al. Risk factors and their combined effects on the incidence rate of subarachnoid hemorrhage--a population-based cohort study. PLoS One 8, e73760, doi:10.1371/journal.pone.0073760 (2013).
3 Knekt, P. et al. Risk factors for subarachnoid hemorrhage in a longitudinal population study. J Clin Epidemiol 44, 933-939 (1991).
4 Sandvei, M. S. et al. Risk factors for aneurysmal subarachnoid hemorrhage - BMI and serum lipids: 11-year follow-up of the HUNT and the Tromso Study in Norway. Acta Neurol Scand 125, 382-388, doi:10.1111/j.1600-0404.2011.01578.x (2012).
5 Fine, J. P. & Gray, R. J. A Proportional Hazards Model for the Subdistribution of a Competing Risk. Journal of the American Statistical Association 94, 496-509 doi:10.1080/01621459.1999.10474144 (1999).
6 Andersson, T., Alfredsson, L., Kallberg, H., Zdravkovic, S. & Ahlbom, A. Calculating measures of biological interaction. Eur J Epidemiol 20, 575-579 (2005).
7 Greenland, S. & Drescher, K. Maximum likelihood estimation of the attributable fraction from logistic models. Biometrics 49, 865-872 (1993).
